# Quantitative evaluation of synthetic MRI for assessing disease activity in thyroid eye disease

**DOI:** 10.1186/s41747-026-00807-x

**Published:** 2026-09-21

**Authors:** Haiyang Zhang, Sien Ping Chew, Duojin Xia, Hui Wang, Yong Zhang, Ling Zhu, Xiaofeng Tao, Yinwei Li, Jing Sun, Mengda Jiang, Huifang Zhou

**Affiliations:** 1 State Key Laboratory of Eye Health, Department of Ophthalmology, Shanghai Ninth People’s Hospital, Shanghai Jiao Tong University School of Medicine, Shanghai, China; 2 School of Health Science and Engineering, University of Shanghai for Science and Technology, Shanghai, China; 3 GE Healthcare, Shanghai, China; 4 Department of Radiology, Shanghai Ninth People’s Hospital, Shanghai Jiao Tong University School of Medicine, Shanghai, China

**Keywords:** Activity assessment, Graves ophthalmopathy, Orbital inflammation, Synthetic magnetic resonance imaging, Thyroid eye disease

## Abstract

**Objective:**

Conventional magnetic resonance imaging (MRI) for thyroid eye disease (TED) is limited by long scan time, restricting quantitative assessment. Synthetic MRI techniques, such as magnetic resonance image compilation (MAGiC), allow for the simultaneous acquisition of T1, T2, and proton density (PD) maps in a single, rapid scan. This study evaluated MAGiC MRI as a quantitative imaging approach for TED disease activity assessment.

**Materials and methods:**

A total of 113 TED patients (49 active, 64 inactive) underwent orbital MRI using a 3-T scanner with MAGiC sequences. Quantitative T1, T2, and PD maps were analyzed for correlations with the clinical activity score (CAS). Image quality of MAGiC_STIR and IDEAL IQ_WP sequences was assessed by two radiologists. Reproducibility was evaluated using intraclass correlation coefficients (ICCs). Logistic regression and receiver operating characteristic (ROC) analyses were performed to identify independent predictors and evaluate diagnostic performance.

**Results:**

CAS showed significant correlations with multiple MAGiC parameters, especially MR_T1 and MR_PD (*p* = 0.018, 0.040, respectively). MAGiC_STIR achieved comparable image quality to IDEAL IQ_WP (64.6% *versus* 67.3%). Quantitative parameters demonstrated excellent reproducibility (ICC = 0.755–0.985). IR_T1, SR_T1, and IR_PD independently predicted TED activity. The combined T1 + PD model achieved the highest accuracy (area under the ROC curve = 0.898; sensitivity = 0.816; specificity = 0.875), and adding T2 did not result in a significant improvement (*p* = 1.000), as assessed by the DeLong test.

**Conclusion:**

Synthetic MAGiC MRI provides fast and reproducible quantitative assessment of TED activity. The T1 + PD model offers an efficient, objective biomarker for disease staging and treatment planning.

**Key Points:**

- ***Question*** Can synthetic MRI provide rapid, reproducible quantitative biomarkers for accurately differentiating active and inactive TED within a single acquisition?
- ***Findings*** Synthetic MRI independently predicted disease activity, and the combined model achieved excellent diagnostic performance.
- ***Relevance Statement*** This study demonstrates that synthetic MRI can effectively assess the disease activity of TED, providing reproducible quantitative biomarkers to enhance disease staging and assist treatment decisions more efficiently.

**Graphical Abstract:**

**Figure:**
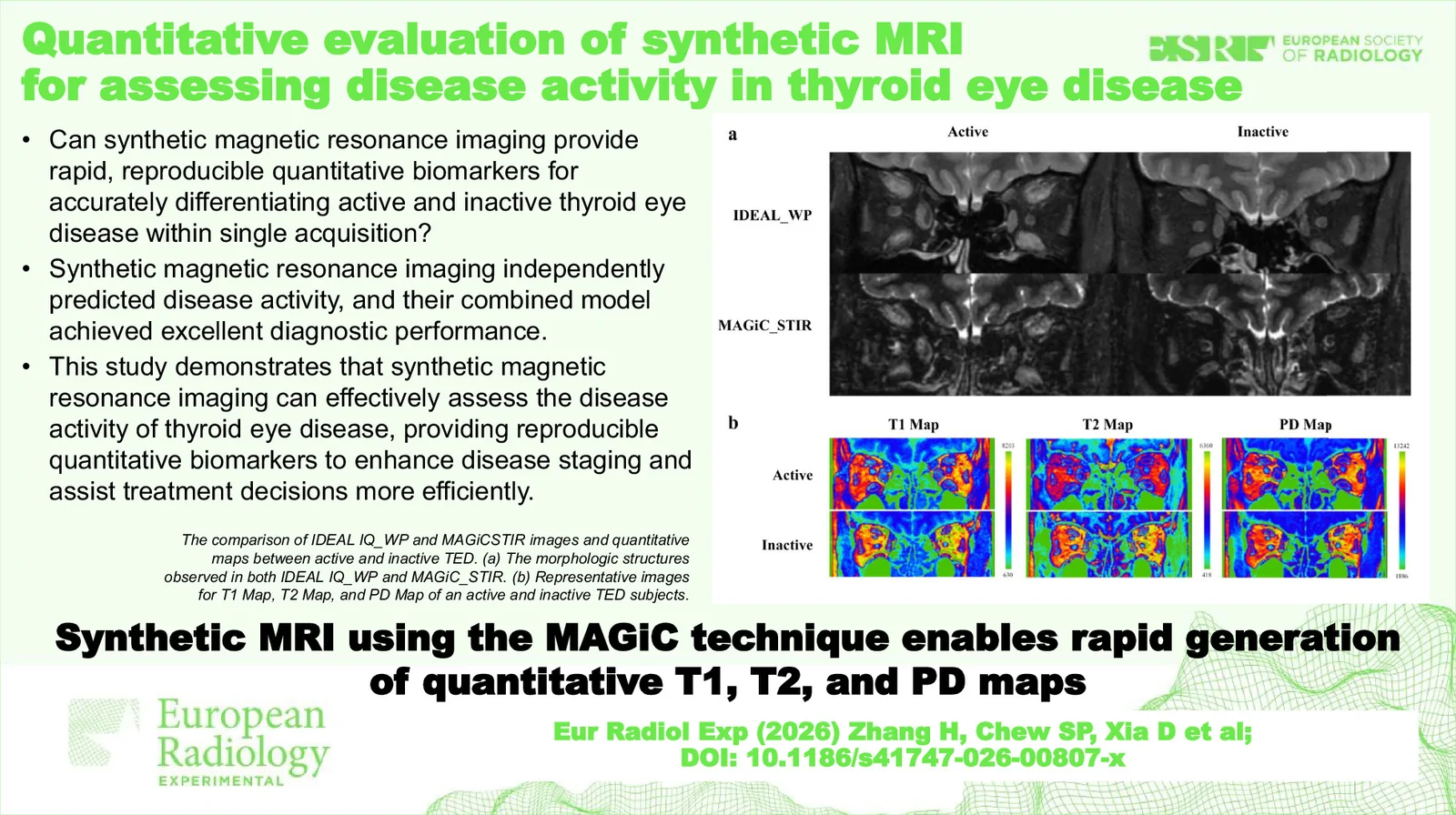

## Background

Thyroid eye disease (TED) is an autoimmune condition primarily associated with Graves’ disease, characterized by inflammation and subsequent fibrosis in orbital tissues [1]. TED typically presents a biphasic clinical course—an active inflammatory stage and a chronic fibrotic stage—requiring precise differentiation for effective management [1]. Accurate staging of TED is crucial as immunosuppressive treatments are most beneficial during the active inflammatory phase, while surgical interventions are reserved for the inactive, fibrotic stage. Misclassification due to inaccurate clinical assessment may lead to delayed initiation of necessary treatments or inappropriate administration of invasive procedures, thus potentially compromising patient outcomes [2]. Currently, the clinical activity score (CAS) based on periocular signs and pain perception is widely utilized for activity assessment [3]. However, CAS has inherent limitations such as inter-observer variability, subjective interpretation, and an inability to identify subclinical or deeper orbital inflammation [4].

Magnetic resonance imaging (MRI) has increasingly become integral for the objective assessment of TED activity, with the capacity to depict inflammatory changes and edema in orbital tissues [5]. Traditional MRI sequences such as “iterative decomposition of water and fat with echo asymmetry and least squares estimation” (IDEAL IQ) have shown utility in activity assessment for TED [6]. Nevertheless, these techniques typically have prolonged scan times and only permit the acquisition of single-parametric data per examination, limiting their clinical practicality [7, 8]. Synthetic MRI techniques such as “magnetic resonance image compilation” (MAGiC) provide a compelling solution by enabling simultaneous quantitative mapping of T1, T2, and proton density (PD) from a single rapid acquisition [9]. MAGiC provides quantitative measurement and significantly reduces imaging time while delivering multiple tissue-characterizing parameters, thereby offering substantial clinical convenience and reproducibility advantages [10].

MAGiC MRI has been previously explored and validated for applications in various body regions, including chest and abdominal imaging, and has demonstrated promising diagnostic utility in head and neck pathologies [10–12]. Consequently, the purpose of our study was to systematically assess the diagnostic performance of MAGiC MRI in activity assessment for TED, aiming to establish a reliable, objective, and clinically applicable imaging biomarker for accurate disease staging and optimized therapeutic decision-making.

## Materials and methods

### Subjects

**Fig. 1:**
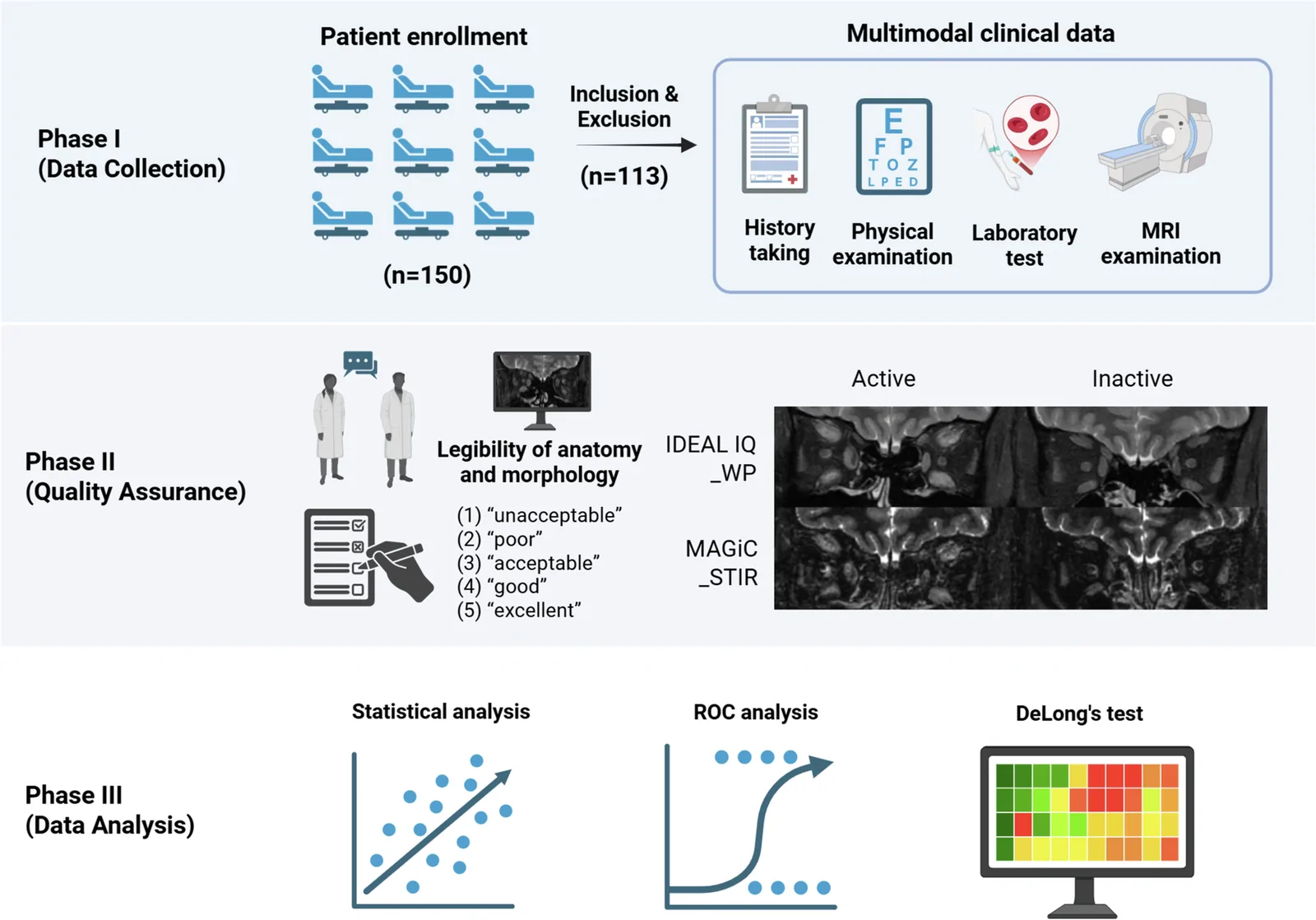
Workflow of the current study. IDEAL IQ, Iterative decomposition of water and fat with echo asymmetry and least-squares estimation quantitation; MAGiC, Magnetic resonance image compilation; MRI, Magnetic resonance imaging; ROC, Receiver operating characteristic; STIR, Short tau inversion-recovery; WP, Water phase

**Fig. 2:**
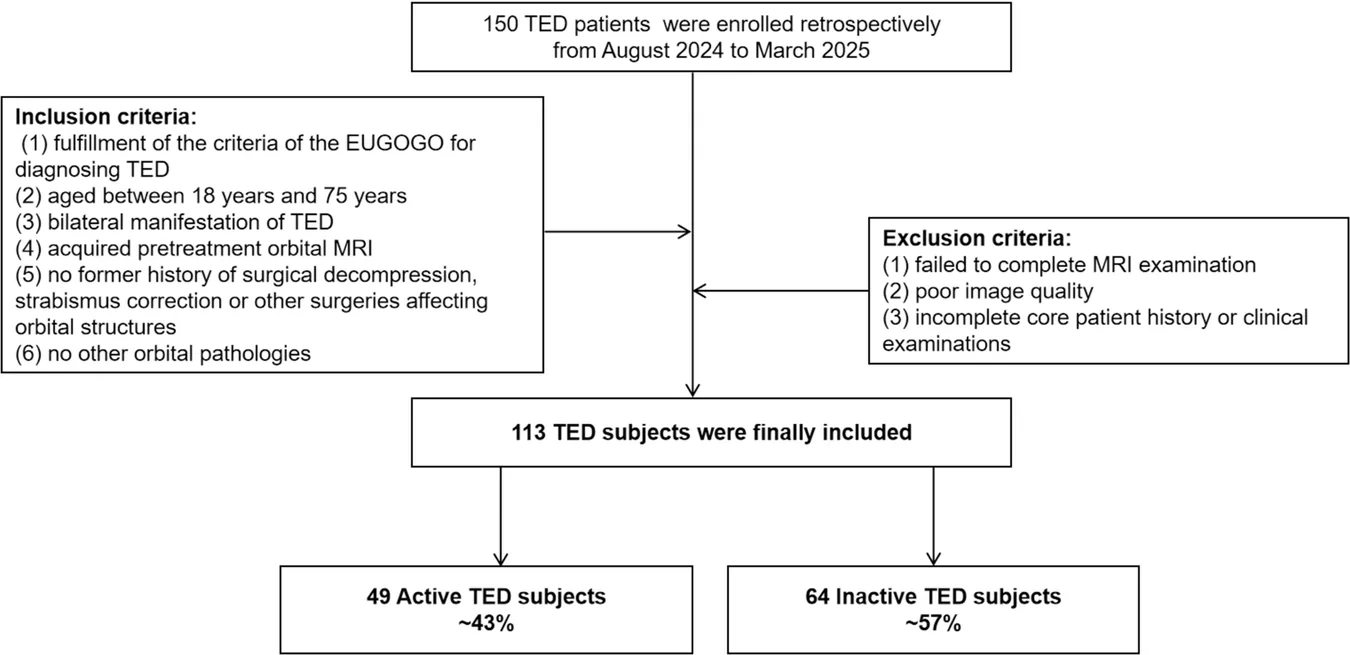
Flowchart for TED patient enrollment from August 2024 to March 2025. EUGOGO, European Group on Graves’ Orbitopathy; TED, Thyroid eye disease

The schematic workflow was outlined and summarized in Fig. 1. The study was conducted in accordance with the Declaration of Helsinki and approved by the hospital institutional ethics committee (SH9H-2023-T406-2). Patients diagnosed with TED between Aug 2024 and March 2025 were retrospectively included for eligibility (Fig. 2). Inclusion criteria consisted of (1) fulfillment of the criteria of the EUGOGO for diagnosing TED [3]; (2) aged between 18 years and 75 years; (3) bilateral manifestation of TED; (4) acquired pretreatment orbital MRI; (5) no former history of surgical decompression, strabismus correction or other surgeries affecting orbital structures; and (6) no other orbital pathologies. Subjects were excluded if they had: (1) failed to complete MRI examination; (2) poor image quality; and (3) incomplete core patient history or clinical examinations.

### Clinical evaluation

For all participants, demographic and clinical data were collected. Ophthalmological examinations such as CAS, proptosis (using the Hertel exophthalmometer [13]) and intraocular pressure were performed. Laboratory examinations were performed to assess thyroid function and antibody status. TED activity was primarily evaluated using MRI and CAS, in accordance with established clinical guidelines and the contemporary updated perspectives [3, 14, 15]. CAS was evaluated by an experienced ophthalmologist, which contains seven parameters including spontaneous retrobulbar pain, pain on attempted upward or lateral gaze, eyelid redness, conjunctival redness, swelling of the caruncle or plica, swelling of the eyelids, and conjunctival edema. Patients with a CAS ≥ 3 and active orbital MRI findings were categorized as having active TED, while those with a CAS < 3 and inactive orbital MRI were categorized as having inactive TED. In cases of discordance between CAS and MRI results, a multidisciplinary team led by an orbital disease specialist with 20 years of experience conducted the adjudication by integrating MRI findings with relevant clinical ophthalmic assessments and laboratory findings, as reported in our previous study [16–18]. Since CAS represents an independent manifestation of orbital lesions and its assessment may be subjective, comprehensive evaluation of multiple disease indicators is essential, with particular emphasis on MRI, which provides direct visualization of pathological lesions. Specifically, cases exhibiting prominent orbital inflammation—manifested by significantly increased T2 signal intensity of enlarged extraocular muscles (EOMs) or lacrimal glands—were classified as active, irrespective of low CAS assessment results. Additionally, progressive and severe subjective symptoms such as retrobulbar pain, diplopia, and photophobia, combined with markedly elevated thyrotropin receptor autoantibody–TRAb levels, were also mainly considered active stage, as these patients would likely benefit from anti-inflammatory therapy. Patients with a high CAS but no evidence of active MRI lesions or other aforementioned clinical activity were mainly considered inactive.

### MRI acquisition and image analysis

To minimize variability, all MRIs were performed on a 3-T MRI scanner (Signa Premier, GE Healthcare, Milwaukee, Wisconsin, USA) with a 48-channel head coil upon its availability. Prior to the examination, subjects were instructed to remove all metal objects from the body, close both eyes naturally, and lie supine on the MRI bed. The imaging protocol included T2 IDEAL and MAGiC. Imaging parameters for T2.

IDEAL technical parameters were as follows: repetition time = 4,500 ms; echo time = 100 ms, flip angle = 111°; echo train length = 24; slice thickness = 3 mm; slice gap = 0.3 mm; and acquisition matrix = 260 × 192. Imaging parameters for MAGiC were as follows: repetition time = 4,500 ms; echo time = 100 ms; flip angle = 103.9°; echo train length = 16; slice thickness = 3 mm; slice gap = 0.3 mm; and acquisition matrix = 256 × 256.

All the quantitative values of this study were obtained *via* GE Healthcare postprocessing software (vendor-provided tool) of the host computer, including T1, T2, and PD values. The regions of interest (ROIs) were created freehand and manually placed on four EOMs (inferior rectus [IR], medial rectus [MR], superior rectus [SR], and lateral rectus [LR]) on the slice of the posterior third of the eyeball by two radiologists with over five years of experience in head and neck radiology, who were blinded to the clinical status of the subjects. Focusing on a consistent posterior segment can avoid regions where boundaries are less stable (*e.g*., near anterior tendons or complex orbital interfaces), thereby improving ROI placement consistency and reducing segmentation ambiguity [19, 20]. The ROI was placed in the T1 map and translated to the T2 and PD maps. Care was taken to avoid inclusion of surrounding fat, vessels, or partial volume effects. For each ROI, values of T1, T2, and PD were extracted and recorded. Finally, T1, T2, and PD values were recorded for the EOMs on both sides to obtain average values, and the final quantitative data were used for further analysis. In our reproducibility assessment, ten subjects were randomly selected from the full cohort, chosen independently of outcomes to represent typical image quality and orbital anatomy encountered in the study population. To evaluate measurement reproducibility, intra- and interobserver agreement were quantified using intraclass correlation coefficients (ICCs). Interobserver variation was assessed by comparing the measurements of the 10 subjects by Operator 1 (the second time) and Operator 2. After the evaluation of ICCs, the measurement results of Operator 1 were used for further analysis.

### Quality assessment for IDEAL IQ_WP and MAGiC_STIR

The quality of representative images for each subject, which included both the water phase (WP) of IDEAL IQ (IDEAL IQ_WP), and the STIR images of MAGiC (MAGiC_STIR) was assessed by two radiologists with over five years of experience in head and neck radiology. IDEAL IQ_WP images were selected as the reference conventional sequence because water-only Dixon reconstruction provides robust fat suppression and highlights edema/inflammatory water components in the orbit, which is clinically relevant for TED assessment. MAGiC_STIR images were generated because STIR is a commonly used fat-suppressed contrast in orbital imaging and is relatively robust to field inhomogeneity, enabling assessment of anatomic legibility and diagnostic usability of MAGiC-derived morphologic images. Although WP and STIR are not identical contrasts, both emphasize water-related inflammatory changes under fat suppression and therefore provide a practical and clinically meaningful basis for image-quality comparison.

The diagnostic image quality and the legibility of key anatomic and morphologic structures were evaluated, according to the established criteria from a previous study [21]. The diagnostic quality of the images was rated on a scale from 1 to 5, with ratings ranging from “unacceptable” (1), “poor” (2), “moderate” (3), “good” (4), and “excellent” (5). Ratings from 3 to 5 were categorized as “acceptable”, and ratings from 1 to 2 were categorized as “unacceptable”. The ratings were based on the overall image quality, considering factors such as spatial resolution, contrast, and visibility of key structures. MAGiC_STIR was compared with conventional IDEAL IQ_WP imaging in terms of these criteria. Additionally, the legibility of critical anatomical structures of EOMs, including the IR, MR, LR, and SR muscles, was also evaluated. The assessment determined whether these structures were clearly identifiable in both imaging modalities. Differences in the performance of the two techniques were analyzed to determine the relative effectiveness of MAGiC_STIR for visualizing key anatomical features associated with TED.

### Statistical analysis

All data analyses were performed using the SPSS software (SPSS Version 26.0, IBM Corp., NY, USA) and GraphPad Prism (GraphPad Version 9.0, GraphPad software). The normality of continuous variables was evaluated using the Shapiro–Wilk test. Data conforming to a normal distribution were expressed as mean ± standard deviation (SD) and compared using an independent samples *t*-test. For non-normally distributed data, the Mann–Whitney *U*-test was employed, with results reported as median (interquartile range [IQR]). Categorical variables were analyzed using Pearson χ² test and presented as frequencies or proportions. Statistical significance was defined as a two-tailed *p* < 0.05. Correlation analyses were performed for CAS and each parameter of EOMs. The univariate analysis with enter selection was used to assess disease activity in TED subjects. A *p* < 0.05 in activity assessment was included in the multivariate analysis. In the multivariate analysis, all parameters with a *p* < 0.05 were simultaneously included in determining the independently predicted indicator. The ICC with 95% confidence intervals (CIs) was calculated to evaluate measurement repeatability. ICC values range from 0 to 1.000, where higher values (closer to 1.000) denote superior reproducibility. Diagnostic performance for disease activity assessment was quantified using receiver operating characteristic (ROC) curve analysis, with optimal cutoff values determined by maximizing the Youden index. Inter-model comparisons were performed using the DeLong test for paired ROC curves, with statistical significance set at *p* < 0.05 (two-tailed). A sensitivity analysis was performed by directly comparing the area under the receiver operating characteristic curve (AUCs) using the DeLong test before and after excluding the discordant cases (*i.e*., CAS and imaging features did not indicate the same activity category) in the same set of overlapping patients.

## Results

### Subject enrollment

A total of 113 TED subjects were included in this study, comprising 49 active and 64 inactive cases. In our cohort, 20 patients had discordant CAS and MRI assessments. Among them, 12 patients had CAS < 3 but demonstrated MRI evidence of active orbital inflammation, and 8 patients had CAS ≥ 3 but lacked MRI features suggestive of active inflammation. After detailed multidisciplinary team discussion, 14 cases were assigned to the final active group, and 6 cases were assigned to the final inactive group according to the prespecified protocol. Baseline demographic and clinical characteristics, as shown in Table 1, were comparable between groups, with no statistically significant differences in age, gender distribution, smoking index, proptosis, or intraocular pressure. The only significant difference was observed in CAS, which was higher in the active group compared with the inactive group (*p* = 0.003).

**Table 1:** Demographic and clinical data of subjects

Variable	Active (*n* = 49)	Inactive (*n* = 64)	*p*
Age (years)	47.2 (46.0–57.4)	45.0 (31.7–58.3)	0.347
Gender (female/male)	21/28	39/25	0.086
Smoking history (yes/no)	6/43	4/60	0.437
CAS	3.76 (2.43–5.09)	2.05 (0.56–3.54)	0.003
Proptosis (mm)	19.59 (18.45–20.73)	19.45 (17.28–21.62)	0.254
IOP (mmHg)	18.25 (16.50–19.12)	17.00 (15.00–19.00)	0.256

*CAS* Clinical activity score, *IOP* Intraocular pressure

### Workflow and efficiency metrics

Retrospective workflow analysis revealed that the MAGiC sequence streamlined image acquisition to 5 min 30 s, providing concurrent T1, T2, and PD mapping within a single scan. This represents a significant time savings compared with traditional multi-sequence protocols, which required approximately 14 min. Additionally, post-processing—including quantitative map generation and manual ROI delineation—took approximately 5–6 min per case for experienced operators.

### Correlation analysis between clinical information and quantitative MRI parameters

**Fig. 3:**
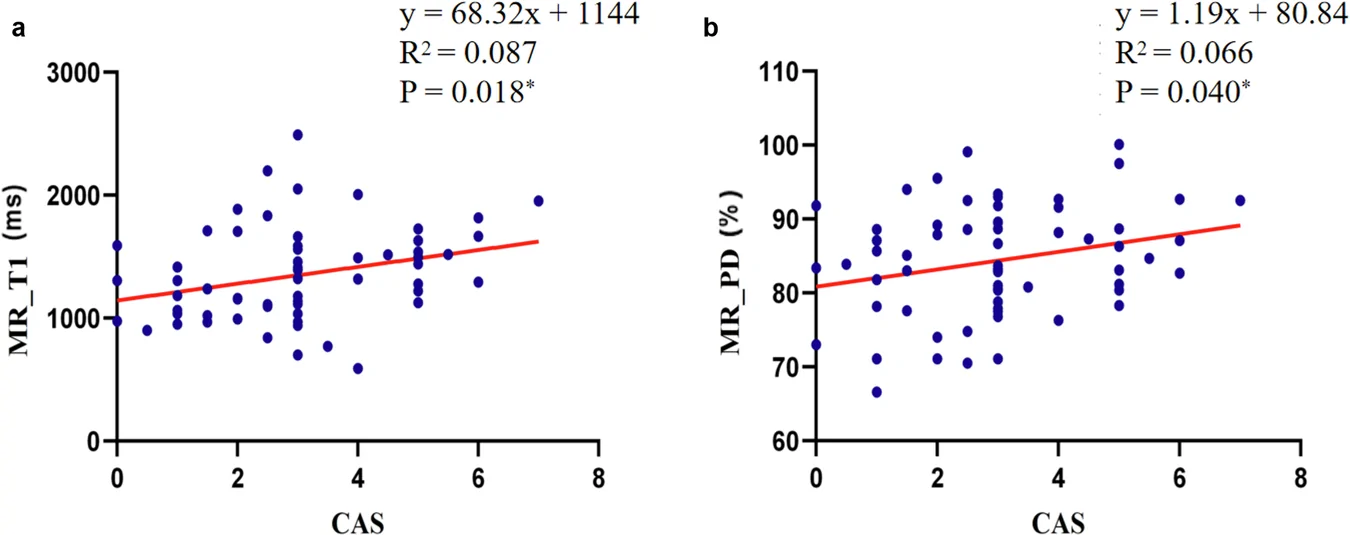
Correlation analysis of CAS and quantitative MRI parameters. **a** MR_T1. **b** MR_PD. CAS, Clinical activity score; MR, Medial rectus; PD, Proton density

Correlation analysis revealed significant associations between the CAS and quantitative MRI parameters of EOMs (Fig. 3). Among all parameters, MR_T1 and MR_PD showed significant correlation with CAS (*p* = 0.018, 0.040, respectively), suggesting the potential of both T1 and PD as sensitive markers of disease activity. These findings indicate that changes in T1 and PD values are consistent with the clinical manifestations of TED.

### Image quality analysis

Excellent intraobserver and interobserver agreement was observed for all quantitative parameters derived from T1, T2, and PD maps, as summarized in Table S1. ICCs for intraobserver reproducibility ranged from 0.755 to 0.985, while interobserver ICCs ranged from 0.842 to 0.981. The highest reproducibility was observed in SR_PD (ICC_intra = 0.985; ICC_inter = 0.981), and all parameters demonstrated ICCs above 0.75, confirming the robustness of ROI-based quantitative measurements in EOMs.

**Fig. 4:**
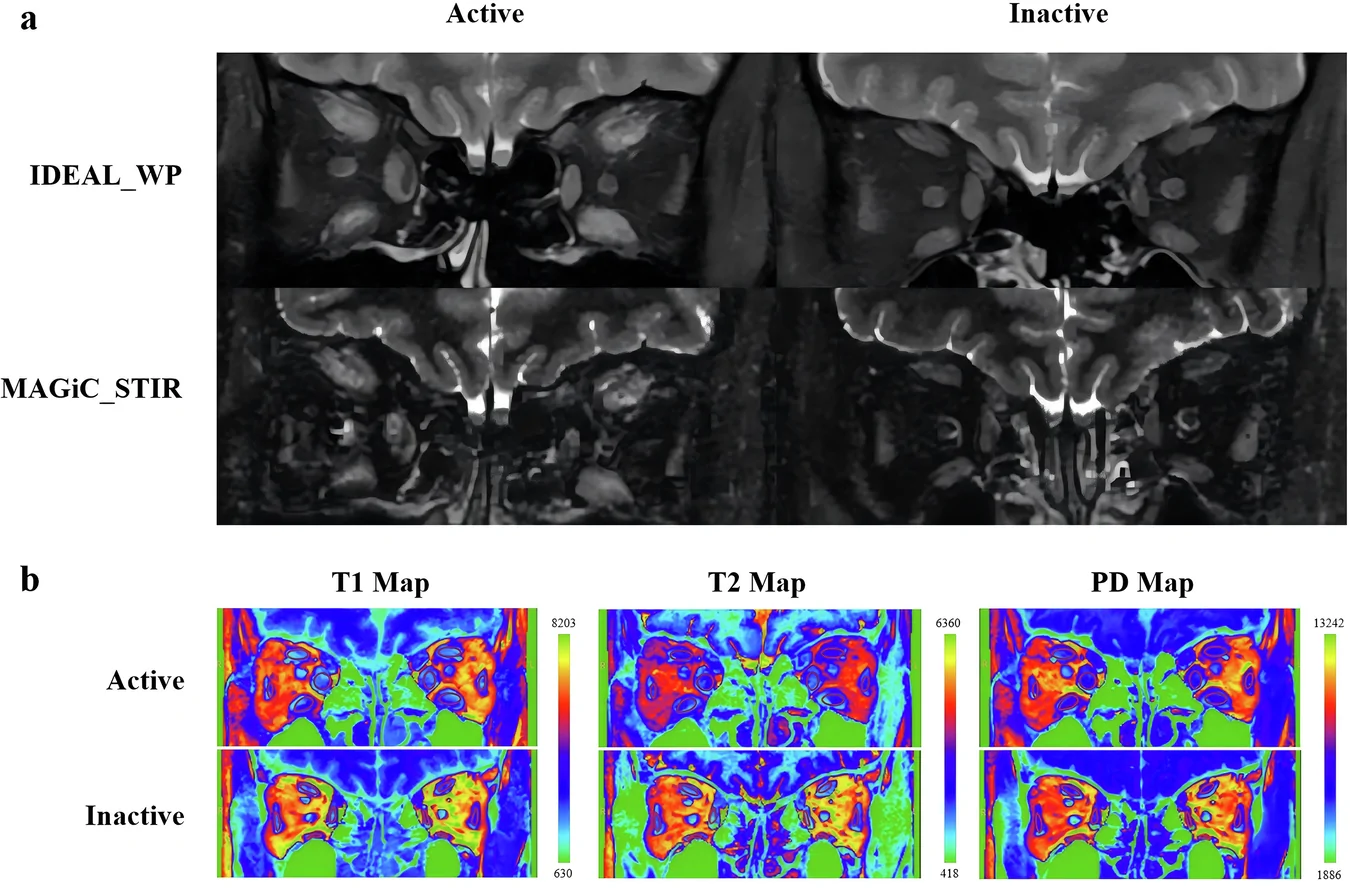
Comparison of IDEAL IQ_WP and MAGiC_STIR images and quantitative maps between active and inactive TED. **a** The morphologic structures observed in both IDEAL IQ_WP and MAGiC_STIR. **b** Representative images for T1 map, T2 map, and PD map, respectively, of an active and inactive TED subject. IDEAL IQ, Iterative decomposition of water and fat with echo asymmetry and least-squares estimation quantitation; MAGiC, Magnetic resonance image compilation; PD, Proton density; STIR, Short tau inversion-recovery; WP, Water phase

The image quality of MAGiC_STIR was evaluated by comparing it to conventional IDEAL IQ_WP imaging. As presented in Table 2, MAGiC_STIR showed a slightly lower but similar acceptable quality rating (64.6%) compared to IDEAL IQ_WP (67.3%). We applied a relatively stringent quality threshold focused on fine anatomic delineation of orbital structures for diagnostic and research purposes; nevertheless, most images still met diagnostic requirements. Anatomic and morphologic structures were analyzed, and the legibility of the images was presented in Table 3, as evaluated using MAGiC_STIR compared to IDEAL IQ_WP imaging. The legibility of the EOMs, including the IR, MR, SR, and LR, in general, was evaluated across both imaging methods as shown in Fig. 4a. For IDEAL IQ_WP imaging, about 90% of the structures were clearly legible in the vast majority of cases. For MAGiC_STIR, about 74% of the structures were clearly legible. The illustrative comparison of T1, T2, and PD maps between active and inactive TED phases (Fig. 4b) visually reinforces our quantitative findings.

**Table 2:** The comparison of the diagnostic quality of IDEAL IQ_WP and MAGiC_STIR

Quality	IDEAL IQ_WP (*n* = 113)	MAGiC_STIR (*n* = 113)
Acceptable range	76 (67.26%)	73 (64.60%)
Unacceptable range	37 (32.74%)	40 (35.40%)

*WP* Water phase, *STIR* Short-T1 inversion restoration, *IDEAL* Iterative decomposition of water and fat with echo asymmetry and least-squares estimation quantitation sequence, *MAGiC* Magnetic resonance image compilation, *IDEAL IQ* Iterative decomposition of water and fat with echo asymmetry and least-squares estimation quantitation

**Table 3:** Legibility of anatomic and morphologic structures

Quality	IDEAL IQ_WP (*n* = 113)				MAGiC_STIR (*n* = 113)			
	IR	MR	SR	LR	IR	MR	SR	LR
Legible	105	104	108	105	79	84	87	86
Illegible	8	9	5	8	34	29	26	27

*WP* Water phase, *STIR* Short-T1 inversion restoration, *EOM* Extraocular muscle, *IR* Inferior rectus, *MR* Medial rectus, *SR* Superior rectus, *LR* Lateral rectus

### The performance of T1, T2, and PD maps in activity assessment

Univariable and multivariable logistic regression (Supplementary Table S2) demonstrated that T1 and PD maps provided the strongest association with TED activity. Specifically, IR_T1, SR_T1, and IR_PD remained independently significant across models, highlighting their diagnostic robustness.

When evaluating combined models, the T1 + PD combination emerged as the most effective, achieving comparable diagnostic performance to the full T1 + T2 + PD model, but without additional complexity (Supplementary Table S3). The inclusion of T2 parameters yielded only marginal improvements and did not significantly enhance predictive power. Overall, these findings indicate that integrating T1 and PD maps offers the most efficient approach for distinguishing active from inactive TED, while T2 plays a supplementary role.

### The performance of combined models in activity assessment

**Fig. 5:**
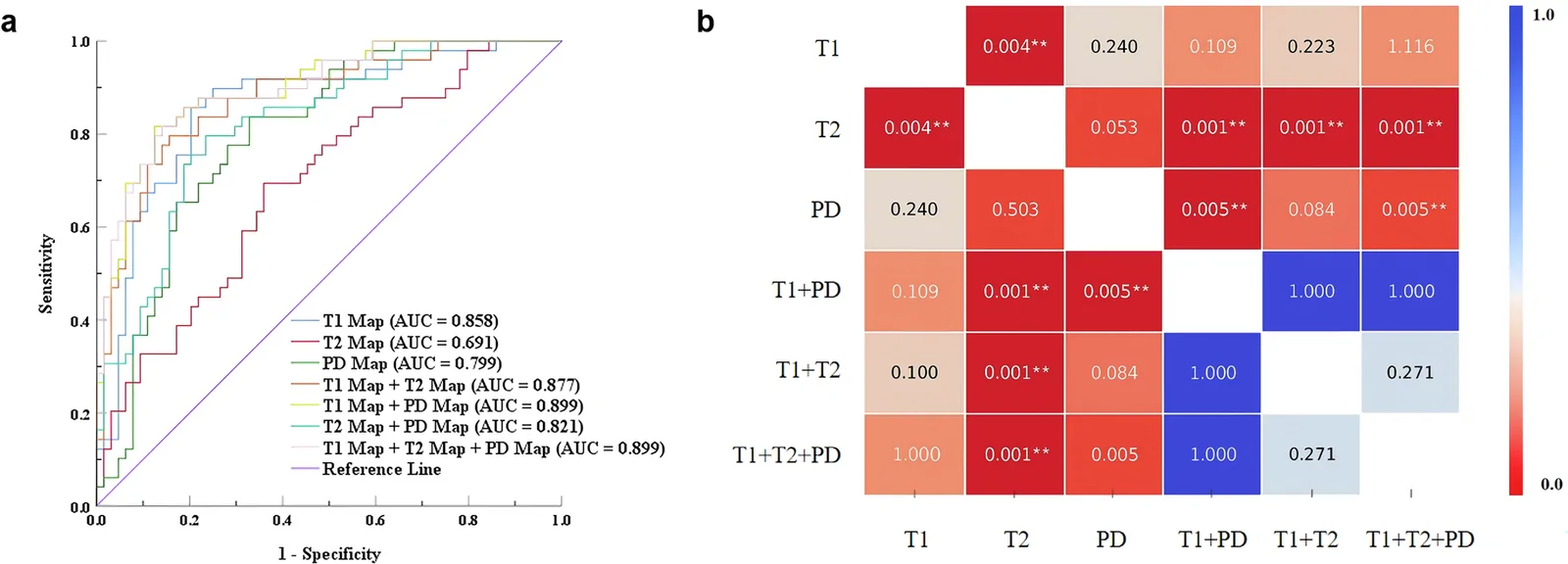
Performance evaluation of MAGiC quantitative models in differentiating disease activity in TED. **a** The ROC curves of different models for evaluating active from inactive TED subjects. **b** The heatmap illustrates the DeLong test results comparing the MAGiC quantitative models. AUC, Area under the curve; PD, Proton density; ROC, Receiver operating characteristic; TED, Thyroid eye disease

MAGiC models demonstrated significant differences in diagnostic performance for activity assessment. The T1 + PD combined model achieved the highest specificity (0.875) and a balanced sensitivity (0.816), with an AUC of 0.898 (Fig. 5a, Table 4, and Supplementary Table S4). Although the full T1 + T2 + PD model attained the numerically highest AUC (0.902), it showed no significant improvement over T1 + PD (DeLong test: *p* = 1.000) (Fig. 5b). Critically, the T2 map alone exhibited the lowest diagnostic accuracy (AUC = 0.724; sensitivity = 0.694, specificity = 0.641) and was significantly outperformed by T1 + PD (*p* < 0.001) (Fig. 5b, Table 4, and Supplementary Table S4). While the PD map provided high sensitivity (0.837), its standalone specificity was suboptimal (0.672), leading to reduced positive predictive value (0.661). These results confirm that T1 + PD is the most efficient combination, offering statistically equivalent accuracy to the full multiparametric model. The sensitivity analysis (Table S5) confirms the robustness of MRI-derived biomarkers for assessing disease activity after excluding discordant cases. The T1 + PD combined model achieved the best fit and highest AUC (0.837), followed by the T1 + T2 + PD combined model with the second highest AUC (0.836). This sensitivity analysis with relatively similar results to our primary result confirms the robustness of MRI-derived biomarkers for assessing disease activity.

**Table 4:** The comparisons of different model performance in TED activity assessment

Quantitative map(s)	Sensitivity	Specificity	PPV	NPV
T1 Map	0.857	0.797	0.764	0.879
T2 Map	0.694	0.641	0.596	0.732
PD Map	0.837	0.672	0.661	0.843
T1 Map + T2 Map	0.796	0.844	0.833	0.862
T1 Map + PD Map	0.816	0.875	0.722	0.831
T2 Map + PD Map	0.796	0.766	0.756	0.900
T1 Map + T2 Map + PD Map	0.816	0.859	0.796	0.844

*NPV* Negative predictive value, *PD* Proton density, *PPV* Positive predictive value, *TED* Thyroid eye disease

## Discussion

This study investigated the diagnostic value of MAGiC to provide objective, reproducible information that complements clinical scoring in the assessment of TED activity, focusing on image quality and quantitative maps. In a previous study, they utilized MAGiC to assess TED activity by using T1, T2, and PD maps of the EOMs as a whole [22]. In contrast, our study analyzed each EOM separately (IR, SR, MR, and LR) and demonstrated that MAGiC offers objective and reproducible markers for evaluating disease activity in individual muscles. In terms of image quality, the MAGiC_STIR images offered sufficient spatial resolution and contrast for accurate delineation of EOMs. Consistent with previous studies, the reproducibility of measurements was excellent, as indicated by high intra- and interobserver ICCs (all > 0.75), confirming the reliability of quantitative map-based evaluations. Regarding the diagnostic performance of individual quantitative maps, both parameters of T1 and PD maps showed stronger associations with CAS than T2. In multivariable logistic regression, specific parameters such as IR_T1, SR_T1, and IR_PD consistently retained statistical significance, while parameters of T2 maps were less robust. Third, the combination of quantitative parameters significantly enhanced diagnostic performance compared to single-parameter models. Notably, while the full model integrating T1, T2, and PD maps provided comprehensive information, the T1 + PD combination achieved comparable diagnostic effectiveness, with overlapping predictive markers and no significant difference in discriminative capacity.

In the active phase, elevated T2 and PD signals dominantly reflect inflammatory edema and increased tissue water content, aligning with the significant associations of IR_T2 and IR_PD with disease activity in our regression models (*p* = 0.023, < 0.001, respectively). Conversely, the inactive phase demonstrates prolonged T1 and reduced PD, consistent with fibrotic tissue remodeling and decreased free water. Notably, the PD map’s dual sensitivity—rising with acute inflammation (active phase) and falling with chronic fibrosis (inactive phase) [9, 23, 24]—highlights its unique role in differentiating TED stages, which traditional T2-weighted imaging alone may fail to capture when pathologies overlap [25, 26]. These findings corroborate the limitations of the CAS, which was described in other studies, particularly in detecting subclinical disease [27]. Most importantly, this study demonstrated the independent diagnostic value of PD mapping in TED—a concept previously explored primarily in neuroimaging [8, 9, 28, 29], where PD serves as a surrogate for tissue hydration. The enhanced performance of combined models can be explained by the biologic basis of TED: active disease is characterized by edema and inflammation (T2↑, PD↑), whereas the inactive phase shows fibrotic remodeling (T1↑, PD↓). Clinically, these findings support the use of MAGiC for accurate and objective staging of TED. The integration of quantitative mapping offers considerable advantages in evaluating equivocal or borderline cases, such as patients with CAS scores between 2 and 3, where clinical uncertainty often complicates therapeutic decision-making. In such scenarios, quantitative imaging markers can serve as adjunctive tools to guide the timely initiation of treatment and avoid unnecessary interventions in inactive disease stages, ultimately improving patient outcomes [5].

**Fig. 6:**
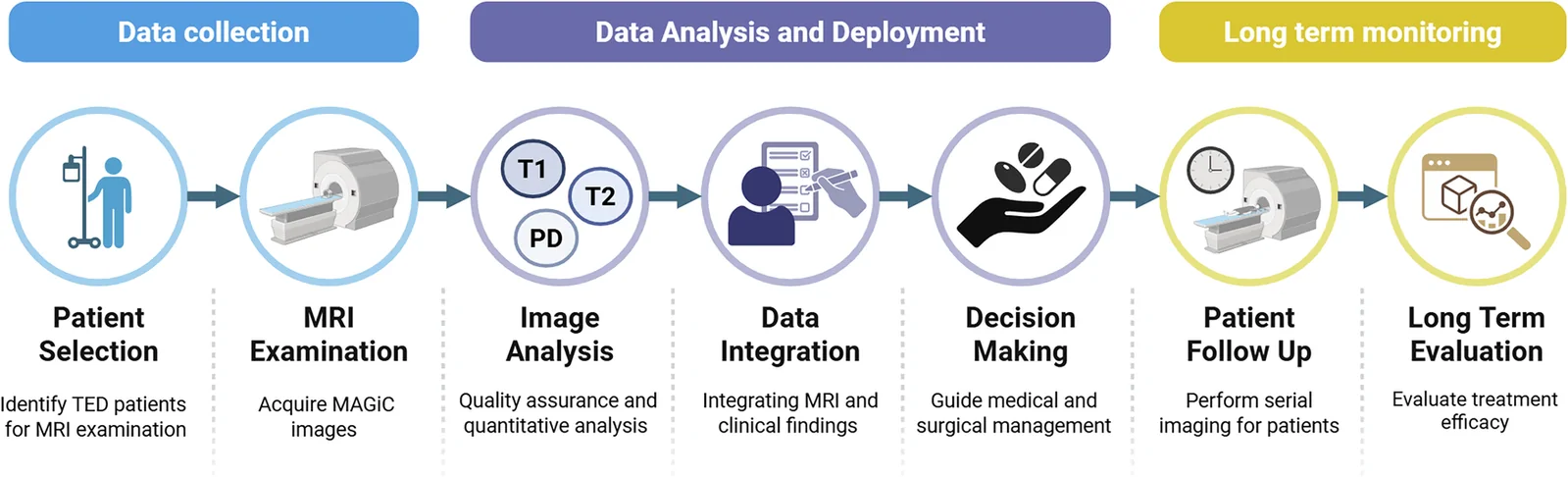
Proposed clinical algorithm for TED management with the application of MAGiC MRI. CAS, Clinical activity score; IV, Intravenous; MAGiC, Magnetic resonance image compilation; MRI, Magnetic resonance imaging; TED, Thyroid eye disease

To facilitate clinical implementation, we propose a streamlined diagnostic algorithm (Fig. 6). In this framework, MAGiC serves as a potential diagnostic tool for patients, especially when anterior signs are insufficient to reflect intraorbital soft tissue inflammation. By leveraging the independent diagnostic value of PD and T1 mapping, clinicians can more reliably identify subclinical inflammation, ensuring that active patients receive timely intervention while preventing inappropriate treatment for those in the fibrotic stage.

From a practical workflow perspective, the clinical value of MAGiC lies not only in its quantitative capability but also in its efficiency [21]. By generating T1, T2, and PD maps in a single acquisition, MAGiC may reduce the need for multiple separate quantitative sequences and shorten the overall examination burden, which is particularly relevant in orbital imaging [30, 31]. In routine practice, this technique may be most useful as an adjunctive tool for patients with equivocal or borderline activity, where conventional clinical scoring alone may be insufficient for confident treatment decision-making. At the same time, its current implementation still depends on adequate image quality, postprocessing availability, and reproducible ROI placement, and therefore should be considered complementary to—rather than a replacement for—standard ophthalmological examination and radiologic interpretation [32, 33].

This study is subject to several limitations. First, it was conducted as a single-center study with the use of a single 3 T GE scanner, which may limit the generalizability of our findings; future confirmatory studies, especially multicenter and multi-vendor prospective validation, should predefine primary endpoints and apply appropriate multiplicity control to reduce the risk of false-positive findings. Second, although interobserver and intraobserver reproducibility was high, the reliance on manual ROI segmentation may result in variability; future studies should investigate automated delineation techniques using deep learning-based segmentation [34–36]. Third, the legibility of images produced by the MAGiC sequence has yet to be improved, possibly through optimization of acquisition parameters and post-processing algorithms. Furthermore, future prospective studies should report workflow-related outcomes (*e.g*., acquisition time, post-processing time, failure rate, and motion/artifact rates) and include external multicenter validation to determine feasibility in real-world practice.

To transition from proof-of-concept to clinical standardization, a structured validation pathway for MAGiC-derived biomarkers is required. First, prospective multi-center studies utilizing various 3-T MRI vendors are necessary to establish robust, platform-independent reference intervals for T1, T2, and PD values in orbital tissues. This will address potential variability in synthetic MRI algorithms across different manufacturers. Second, longitudinal studies should be used to assess the predictive value of these biomarkers for treatment outcomes, specifically monitoring how PD and T1 alterations mirror the resolution of edema following immunomodulatory therapy. Finally, future research incorporating histopathological correlation remains important. By correlating surgical specimen findings such as cellular density, collagen deposition, and inflammatory infiltration with *in vivo* MAGiC-derived parameters, researchers can elucidate the exact microstructural changes that drive alterations in T1, T2, and PD values. This cross-validation will establish a solid pathological foundation for synthetic MRI, elevating these quantitative metrics from empirical observations to mechanistically understood, robust clinical biomarkers.

In conclusion, our study demonstrated that MAGiC, integrating quantitative T1, T2, and PD maps, significantly improves the discrimination between active and inactive TED compared to conventional approaches. The T1 + PD model showed the highest diagnostic accuracy; the T1 + T2 + PD model also demonstrated strong performance, although the inclusion of T2 provided only marginal improvements. These findings support the potential of MAGiC and its clinical utility for TED management. Future research should explore the role of these biomarkers in monitoring treatment response and long-term disease progression.

## Supplementary information

**Additional file 1:** **Table S1** The evaluation of reproducibility using intraclass correlation coefficient. **Table S2** Binary logistic regression analysis for MAGiC quantitative models. **Table S3** Multivariable logistic regression analysis for combined models. **Table S4** Comparisons of different combined models using MAGiC quantitative maps. **Table S5** Sensitivity analysis with direct DeLong’s test on overlap patients.

## Data Availability

Available on request.

## Abbreviations

AUC: Area under the receiver operating characteristic curve
CAS: Clinical activity score
EOM: Extraocular muscle
ICC: Intraclass correlation coefficient
IDEAL: Iterative decomposition of water and fat with echo asymmetry and least-squares estimation
IR: Inferior rectus
LR: Lateral rectus
MAGiC: Magnetic resonance image compilation
MR: Medial rectus
MRI: Magnetic resonance imaging
PD: Proton density
ROC: Receiver operating characteristic
ROI: Region of interest
SR: Superior rectus
STIR: Short tau inversion-recovery
TED: Thyroid eye disease
WP: Water phase
