# Supplementary material for: Quantitative evaluation of synthetic MRI for assessing disease activity in thyroid eye disease
Source: Eur Radiol Exp. 2026 Sep 21;10:139. doi: 10.1186/s41747-026-00807-x (PMC13594039; doi:10.1186/s41747-026-00807-x)
Supplement: Supplementary file 1 [file 41747_2026_807_MOESM1_ESM.pdf]

Quantitative evaluation of synthetic MRI for assessing disease activity in thyroid eye disease

ELECTRONIC SUPPLEMENTARY MATERIAL

Table S1 The evaluation of reproducibility using intraclass correlation coefficient

**Parameters**	**ICC (95% CI)**	
	**Inter-operator**	**Intra-operator**
**IR_T1**	0.882 (0.858–0.921)	0.886 (0.857–0.917)
**IR_T2**	0.922 (0.898–0.970)	0.972 (0.935–0.990)
**IR_PD**	0.895 (0.859–0.927)	0.767 (0.750–0.798)
**MR_T1**	0.881 (0.853–0.924)	0.771 (0.752–0.796)
**MR_T2**	0.852 (0.818–0.889)	0.755 (0.752–0.783)
**MR_PD**	0.905 (0.884–0.944)	0.952 (0.916–0.977)
**SR_T1**	0.855 (0.817–0.894)	0.937 (0.912–0.964)
**SR_T2**	0.964 (0.916–0.993)	0.959 (0.919–0.983)
**SR_PD**	0.981 (0.951–0.995)	0.985 (0.959–0.993)
**LR_T1**	0.842 (0.801–0.864)	0.942 (0.897–0.965)
**LR_T2**	0.942 (0.925–0.986)	0.861 (0.816–0.884)
**LR_PD**	0.877 (0.851–0.901)	0.937 (0.888–0.971)

ICC, intraclass correlation coefficient; IR, inferior rectus; MR, medial rectus; SR, superior rectus; LR, lateral rectus; PD, proton density.

Table S2 Binary logistic regression analysis for MAGiC quantitative models

**Map**	**Parameter**	**Univariable**				**Multivariable**			
		**B**	**SE**	**OR (95% CI)**	**P**	**B**	**SE**	**OR (95% CI)**	**P**
**T1 map**	IR_T1 (ms)	0.003	0.001	1.003 (1.001–1.004)	**<0.001*****	0.002	0.001	1.002 (1.001–1.003)	**0.007****
	MR_T1 (ms)	0.003	0.001	1.003 (1.002–1.005)	**<0.001*****	0.002	0.001	1.002 (1.000–1.003)	**0.034***

	SR_T1 (ms)	0.002	0.001	1.002 (1.001–1.004)	**<0.001*****	0.002	0.001	1.002 (1.001–1.003)	**0.005****
	LR_T1 (ms)	0.000	0.000	1.000 (1.000–1000)	0.541				
T2 map	IR_T2 (ms)	-0.006	0.002	0.994 (0.989–0.999)	**0.013***	-0.006	0.002	0.994 (0.990–0.999)	**0.023***
	MR_T2 (ms)	0.002	0.002	1.002 (0.997–1.007)	0.393				
	SR_T2 (ms)	0.056	0.018	1.057 (1.021–1.095)	**0.002****	0.053	0.018	1.054 (1.017–1.093)	**0.004****
	LR_T2 (ms)	0.016	0.017	1.016 (0.984–1.050)	0.334				
PD map	IR_PD (%)	0.072	0.019	1.075 (1.035–1.116)	**<0.001*****	0.058	0.018	1.060 (1.024–1.098)	**<0.001*****
	MR_PD (%)	0.083	0.031	1.087 (1.022–1.156)	**0.008****				
	SR_PD (%)	0.074	0.025	1.077 (1.026–1.131)	**0.003****				
	LR_PD (%)	0.044	0.017	1.045 (1.010–1.080)	**0.010***				

MAGiC, magnetic resonance image compilation; T1, longitudinal relaxation time; T2, transverse relaxation time; PD, proton density; IR, inferior rectus; MR, medial rectus; SR, superior rectus; LR, lateral rectus; SE, standard error; OR, odds ratio; CI, confidence interval.

* Statistical significance is indicated by P < 0.05; ** is indicated by P < 0.01; *** is indicated by P < 0.001.

Table S3 Multivariable logistic regression analysis for combined models

Model combination	Parameter	Multivariable			
		B	SE	OR (95% CI)	P
T1 map + T2 map	IR_T1 (ms)	0.002	0.001	1.002 (1.001–1.003)	**0.007****
	IR_T2 (ms)	-0.008	0.004	0.992 (0.985–0.999)	**0.027***

	MR_T1 (ms)	0.002	0.001	1.002 (1.000–1.003)	0.085
	SR_T1 (ms)	0.002	0.001	1.002 (1.000–1.003)	**0.017***
	SR_T2 (ms)	0.034	0.025	1.034 (0.984–1.087)	0.184
**T1 map + PD map**	IR_T1 (ms)	0.002	0.001	1.002 (1.000–1.003)	**0.043***
	IR_PD (%)	0.054	0.021	1.055 (1.012–1.100)	**0.012***
	MR_T1 (ms)	0.001	0.001	1.001 (0.999–1.004)	0.240
	MR_PD (%)	0.040	0.058	1.040 (0.929–1.165)	0.495
	SR_T1 (ms)	0.002	0.001	1.002 (1.000–1.003)	**0.024***
	SR_PD (%)	0.024	0.033	1.024 (0.961–1.092)	0.461
	LR_PD (%)	-0.036	0.025	0.965 (0.919–1.013)	0.152
**T2 map + PD map**	IR_T2 (ms)	-0.001	0.003	0.999 (0.993–1.004)	0.696
	IR_PD (%)	0.050	0.019	1.051 (1.012–1.091)	**0.010****
	MR_PD (%)	0.056	0.042	1.057 (0.974–1.147)	0.184
	SR_T2 (ms)	0.043	0.022	1.044 (1.000–1.090)	0.050
	SR_PD (%)	0.051	0.028	1.052 (0.996–1.111)	0.069
	LR_PD (%)	0.005	0.020	1.005 (0.966–1.046)	0.786
**T1 map + T2 map + PD map**	IR_T1 (ms)	0.002	0.001	1.002 (1.000–1.004)	**0.018***
	IR_T2 (ms)	-0.007	0.005	0.993 (0.984–1.002)	0.146
	IR_PD (%)	0.036	0.024	1.036 (0.988–1.086)	0.140
	MR_T1 (ms)	0.001	0.001	1.001 (0.999–1.004)	0.312
	MR_PD (%)	0.039	0.059	1.040 (0.926–1.167)	0.508
	SR_T1 (ms)	0.001	0.001	1.001 (1.000–-1.003)	0.071
	SR_T2 (ms)	0.031	0.029	1.032 (0.975–1.092)	0.280
	SR_PD (%)	0.039	0.033	1.040 (0.974–1.110)	0.242
	LR_PD (%)	-0.038	0.025	0.963 (0.917–1.012)	0.138

T1, longitudinal relaxation time; T2, transverse relaxation time; PD, proton density; IR, inferior rectus; SR, superior rectus; SE, standard error; OR, odds ratio; CI, confidence interval.

* Statistical significance is indicated by $P < 0.05$; ** is indicated by $P < 0.01$.

Table S4 Comparisons of different combined models using MAGiC quantitative maps

**Model Comparison**	**P**
T1 map vs. T2 map	**0.011***
T1 map vs. PD map	0.599
T1 map vs. T1 map + T2 map	0.745
T1 map vs. T1 map + PD map	0.112
T1 map vs. T2 map + PD map	0.698
T1 map vs. T1 map + T2 map + PD map	0.181
T2 map vs. PD map	**0.023***
T2 map vs. T1 map + T2 map	**<0.001*****
T2 map vs. T1 map + PD map	**<0.001*****
T2 map vs. T2 map + PD map	**0.005****
T2 map vs. T1 map + T2 map + PD map	**<0.001*****
PD map vs. T1 map + T2 map	0.449
PD map vs. T1 map + PD map	**0.021***
PD map vs. T2 map + PD map	0.585
PD map vs. T1 map + T2 map + PD map	**0.026***
T1 map + T2 map vs. T1 map + PD map	0.151
T1 map + T2 map vs. T2 map + PD map	0.531
T1 map + T2 map vs. T1 map + T2 map + PD map	0.146
T1 map + PD map vs. T2 map + PD map	**0.043***
T1 map + PD map vs. T1 map + T2 map + PD map	0.501

T2 map + PD map vs. T1 map + T2 map + PD map	**0.042***

MAGiC, magnetic resonance image compilation; T1, longitudinal relaxation time; T2, transverse relaxation time; PD, proton density.

* Statistical significance is indicated by P < 0.05; ** is indicated by P < 0.01; *** is indicated by P < 0.001.

Table S5 Sensitivity analysis with direct DeLong's test on overlap patients

**Model**	**AUC, n=93**	**AUC, n=113**	**Difference**	**P**
T1 map	0.831	0.804	+0.028	0.6282
T2 map	0.756	0.756	+0.000	nan
PD map	0.789	0.789	+0.000	nan
T1 map + T2 map	0.827	0.813	+0.014	0.8111
T1 map + PD map	0.833	0.838	-0.005	0.9301
T2 map + PD map	0.817	0.818	-0.001	0.8416
T1 map + T2 map + PD map	0.828	0.843	-0.014	0.8115

AUC, Area under the curve; PD, proton density; nan, undefined numerical value.
